# SARS-CoV-2 Reverse Zoonoses to Pumas and Lions, South Africa

**DOI:** 10.3390/v14010120

**Published:** 2022-01-11

**Authors:** Katja Natalie Koeppel, Adriano Mendes, Amy Strydom, Lia Rotherham, Misheck Mulumba, Marietjie Venter

**Affiliations:** 1 Department of Production Animal Studies, Faculty of Veterinary Science, University of Pretoria, Onderstepoort, Pretoria 0110, South Africa; katja.koeppel@up.ac.za; 2 Centre for Veterinary Wildlife Studies, Faculty of Veterinary Sciences, University of Pretoria, Onderstepoort, Pretoria 0001, South Africa; 3 Zoonotic Arbo- and Respiratory Virus Program, Centre for Viral Zoonoses, Department for Medical Virology, University of Pretoria, Pretoria 0110, South Africa; adriano.mendes288@gmail.com (A.M.); aimster.strydom@gmail.com (A.S.); 4 Onderstepoort Veterinary Research Institute, Agricultural Research Council, Onderstepoort, Pretoria 0110, South Africa; RotherhamL@arc.agric.za (L.R.); MulumbaM@arc.agric.za (M.M.)

**Keywords:** SARS-CoV-2, reverse zoonosis, wildlife

## Abstract

Reverse-zoonotic infections of severe acute respiratory syndrome-related coronavirus 2 (SARS-CoV-2) from humans to wildlife species internationally raise concern over the emergence of new variants in animals. A better understanding of the transmission dynamics and pathogenesis in susceptible species will mitigate the risk to humans and wildlife occurring in Africa. Here we report infection of an exotic puma (July 2020) and three African lions (July 2021) in the same private zoo in Johannesburg, South Africa. One Health genomic surveillance identified transmission of a Delta variant from a zookeeper to the three lions, similar to those circulating in humans in South Africa. One lion developed pneumonia while the other cases had mild infection. Both the puma and lions remained positive for SARS-CoV-2 RNA for up to 7 weeks.

## 1. Introduction

Severe acute respiratory syndrome-related coronavirus 2 (SARS-CoV-2) is the causative agent of the disease COVID-19, which has caused a pandemic unlike any the present generation has seen before. SARS-CoV-2 belongs to the Coronaviridae family of positive sense single stranded RNA viruses [1]. The family consists of 46 species of virus, the majority of which have been isolated from animals [2]. Only seven viruses (NL63, 229E, HKU1, OC43, SARS, MERS and SARS-CoV-2) are known to infect humans but each are believed to have a zoonotic origin [3]. For this reason, as well as the high level of sequence similarity to a virus isolated for *Rhinolophus affinis* bats, RaTG13, it is believed that SARS-CoV-2 either descended directly from bats or evolved in a yet to be identified intermediate animal reservoir host before it was transmitted to humans [4,5]. The likely zoonotic spill-over highlights the importance of investigating transmission dynamics in animals to identify susceptible hosts but also define the risk for reverse zoonoses from humans and subsequent evolution.

Investigations of susceptibility of animals to SARS-CoV-2 can be categorised into three groups: those with predicted susceptibility, those experimentally infected, and naturally infected animals, with infections believed to occur through reverse zoonotic events. Studies which predict the susceptibility of animal species primarily utilise bioinformatics methods based on angiotensin converting enzyme 2 (ACE2) sequence homology, which is the receptor for the virus, and the SARS-CoV-2 Spike glycoprotein interaction [6,7,8,9]. Animals which have been successfully infected experimentally include domestic cats [10,11], ferrets [12,13], Rhesus macaques [14,15], fruit bats [13], golden Syrian hamsters [16,17] and deer mice [18]. Animal surveillance programs have also discovered reverse zoonotic events in domestic species including cats [19,20] and dogs [19], as well as captive wildlife populations such as mink [21], otter [22], ferrets [23], lions [24,25], tigers [24], snow leopards, gorillas [22,26], and white-tailed deer [27]. With the emergence of SARS-CoV-2 variants of concern, the question as to whether evolution of the virus will favour reverse-zoonoses and animal transmission is important to address.

South Africa experienced three waves of infection of COVID-19 from March 2020 to October 2021. Wave 1 was characterised by a mixture of the original strains, wave 2 by the beta variant and wave 3 by the Delta variant [28,29]. South Africa has a lucrative wildlife industry mainly based on conservation and tourism with several large feline species being kept in wildlife reserves but also in zoos across the country. Here we describe the natural infection of SARS-CoV-2 in a puma during the first wave and three lions during the third wave in a private zoo in South Africa in at least three transmission events from their handlers. The three lions were all infected with the Delta variant while the puma was infected during the first wave but not genetically investigated. With lions and other big cats being found naturally in wildlife reserves as well as higher density settings in South Africa, the risk to these animals being infected from humans either through close contact or through handling of food requires further attention. It is also equally important to assess the risk of subsequent transmission between animals and prolonged shedding that may give rise to new variants.

## 2. Materials and Methods

### 2.1. Ethics Statement

The study was approved by the Human and Animal Ethics Committee of the University of Pretoria (REC150-20) and Section 20 application by the Department of Agriculture Land Reform and Rural development of South Africa (12/11/1/1/8 (1612 AC)).

### 2.2. Outbreak Description

In July 2020, two pumas (LPZ0017 and LPZ0018) in a private zoo showed signs of anorexia, diarrhoea, and nasal discharges. The two pumas were in one enclosure. LPZ0018 also developed ocular discharge and a dry cough, which persisted for 13 days. LPZ0018 was anaesthetised with medetomidine (2 mg, Kyron Laboratories, Johannesburg, South Africa) and zolazepam and tiletamine (Zoletil^®^, Virbac, South Africa, 40 mg) on 27 July 2020. A nasopharyngeal sample (NP) was taken for SARS-CoV-2 after 5 days of persistent coughing, which did not respond to antibiotic therapy (12 mg/kg bid, Amoxycillin/Clavulanic acid, Auro Amoxiclav, Aurobindo, Johannesburg, South Africa). Follow-up samples were taken on the 13 August, 25 August, and 9 September 2020 of LPZ0018. As puma LPZ0017 only presented with mild clinical signs, it was decided not to anaesthetise the animal for testing purposes. Both pumas made a full recovery after 23 days.

**Figure 1:**
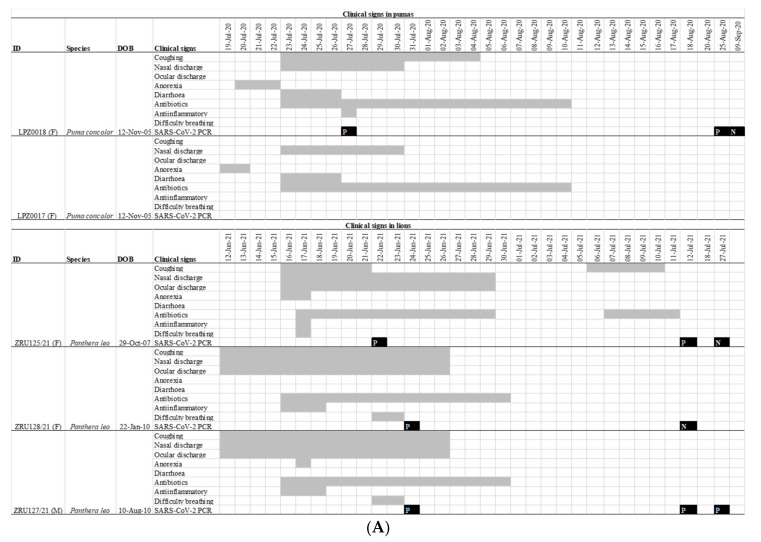

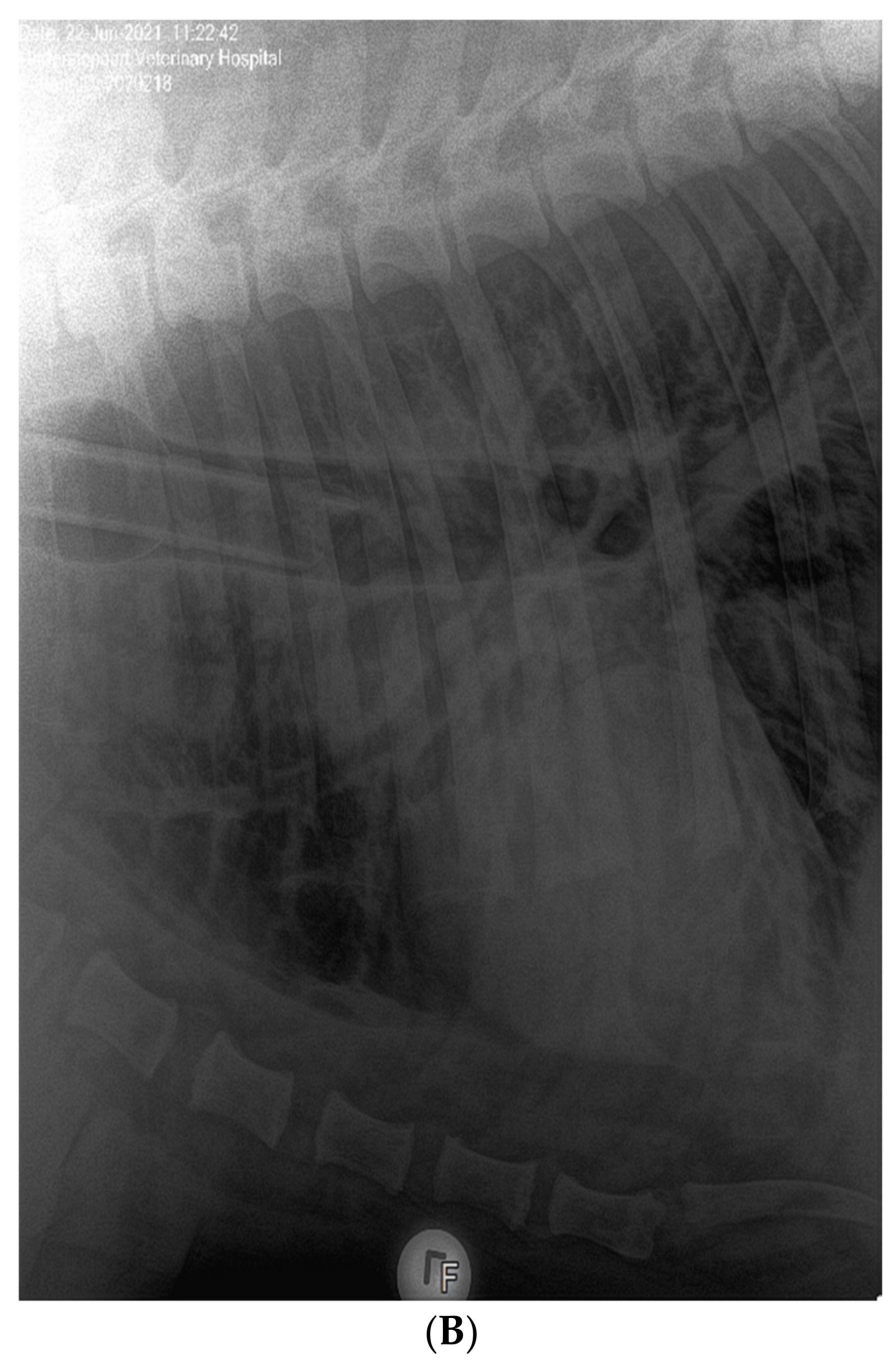
(**A**): Clinical features and timeline of SARS-CoV-2 infection in the pumas and lions. Grey bars indicate the duration of a sign of infection. Black squares indicate the date of a RT-PCR test with a P indicating a positive test and N a negative one. All RT-PCR results shown are for nasopharyngeal swabs. (**B**): Lateral view of the chest of the 14-year-old lion ZRU125/21 showing marked bronchial lung pattern suggestive of bronchopneumonia.

In June 2021, three lions (ZRU125/21, ZRU127/21 and ZRU128/21) who were all born in captivity and raised in a zoo exhibited respiratory symptoms. ZRU127/21 and ZRU128/21 were kept in one enclosure and ZRU125/21 was kept in a separate enclosure. The clinical signs in these lions were predominantly upper respiratory with nasal and ocular discharge and a dry cough for up to 14 to 15 days. A persistent cough was seen between 5 and 15 days with worsening and difficulty breathing in two lions (ZRU127/21 and ZRU128/21) for 10 days after the onset of cough. Transient anorexia (1 to 2 days) was seen in 2 out of 3 lions (ZRU125/21 and ZRU127/21). Lions were treated orally with amoxiclav (8 mg/kg bid) and a NSAID (meloxicam, 0.05 mg/kg, qd, coxflam, Novartis, Johannesburg, South Africa). ZRU125/21 did not respond to antibiotics (Amoxycillin/Clavulanic acid, Auro Amoxiclav, Aurobindo, Johannesburg, South Africa). The lioness was immobilized with medetomidine (6 mg) and zolazepam and tiletamine (Zoletil^®^, 100 mg) and a NP sample was tested for SARS-CoV-2 on 22 June. Subsequent oropharyngeal or NP samples were taken on 25 June of the other two lions as well as zoo staff who had direct or indirect contact with the lions (Supplementary Table S1). Staff and lions were monitored in the subsequent weeks for the presence of SARS-CoV-2. Voided faecal samples from the lions were also collected from 25 June to 12 July 2021 (Supplementary Table S2). ZRU125/21 received a dose of dexamethasone (Kortico, Bayer, Johannesburg, South Africa) intravenously as she started to develop pneumonia indicated by bronchial changes on radiographs (Figure 1B). All three lions made a full recovery within 15 to 25 days.

### 2.3. RT-PCR of SARS-CoV-2

Dry nasal swabs were placed into 1 ml of PBS and left at room temperature for 10–30 min. Thereafter the samples were vortexed for 1 min. Nucleic acid was extracted from NP or faecal samples with the Qiamp Viral Mini Kit (Qiagen, Hilden, Germany) according to the manufacturer’s instructions.

The AllpexTM 2019-nCoV assay was used to test for the presence of SARS-CoV-2 in the puma samples at the Agricultural Research Council, Onderstepoort Veterinary Research Institute. Briefly, 8 uL of extract was mixed with 5 uL reaction buffer, 5 uL water, 5 mL of primer and probe mixture, and 2 uL of enzyme mix. The samples were run on a Biorad Cfx96 (Biorad, Hercules, CA, USA) or a Rotor-Gene Q 5plex Platform (Qiagen, Montgomery, MD, USA). Results were analysed using CFX Manager Software (Biorad, Hercules, CA, USA) or Q-Rex Software (Qiagen, Montgomery, MD, USA).

LightMix SarbecoV E-gene and RdRp gene kits (TIP-MOLBIOL, Berlin, Germany) were used to test for the presence of SARS-CoV-2 in lion samples at the Zoonotic, Arbo- and Respiratory Virus research program, Centre for Viral Zoonoses, University of Pretoria. Briefly, 10 uL of template was mixed with 0.5 uL parameter specific reagent, 0.8 uL AgPath-ID™ One-Step RT-PCR Reagents (Applied Biosystems™, Waltham, MA, USA), and 10 uL buffer. Reactions were run on a ViiA7 (Thermo Fisher Scientific, Waltham, MA, USA). Results were analysed using QuantStudio Real-Time PCR Software (Thermo Fisher Scientific, Waltham, MA, USA). The AllpexTM 2019-nCoV assay and LightMix SarbecoV E-gene and RdRp gene kits are approved by the South African Health Products Regulatory Authority (SAHPRA).

### 2.4. Whole Genome Sequencing and Phylogenetic Analysis

Nucleic acid was extracted from 200 uL NP samples with the Qiamp Viral Mini Kit (Qiagen) according to the manufacturer’s instructions in a total elution volume of 50 uL. The RNA clean and concentrator 5 kit (Zymo Research, Irvine, CA, USA) was used to concentrate RNA to a final volume of 15 uL. Ct values were determined with the LightMix SarbecoV E-gene kit using 3 uL RNA and 7 uL nuclease free water. Complementary DNA was synthesized with Superscript IV Reverse Transcriptase (Thermo Fisher Scientific, Waltham, MA, USA) and random primers according to the manufacturer’s instructions. The SARS-CoV-2 genomes were amplified with a 1:1 combination of the Artic primer pools V2 and V3 (https://artic.network/ncov-2019 (accessed on 14 July 2021)). The reaction was done with Q5 High fidelity polymerase (New England Biolabs, Ipswich, MA, USA). PCR clean-up was done with Agencourt AMPure XP beads (Beckman Coulter, Carlsbad, CA, USA) according to the manufacturer’s instructions. Libraries were prepared with the Illumina DNA prep kit (Illumina, Berlin, Germany) using between 100 and 500 ng of cDNA. Tagmentation was done at 55 °C for 15 min and amplification of tagmented DNA was done with the enhanced PCR mix and index adapters. Libraries were purified and size selected with the sample purification beads and 80% ethanol. Libraries were normalized to a final concentration of 12 pM before sequencing using an iSeq 100 i1 Reagent v2 (300 cycles) kit.

FASTQ files were uploaded to the Galaxy web platform and the public server at usegalaxy.eu to analyse the data [30] The Galaxy workflow for the analysis of Illumina paired end sequenced ARTIC amplicon data was used to assemble raw data [30].

### 2.5. Sanger Sequencing of the Spike Gene

cDNA was synthesized from RNA with Superscript III Reverse Transcriptase (Invitrogen™, Waltham, MA, USA) and 5 uM of SARS2_S R1 (GGAGACACTCCATAACACTTAA). First round amplification was done with 10 uM of SARS2_S_F1 (GTCTCTAGTCAGTGTGTTA) and 10 uM SARS2_S_R1 and the Platinum™ II Taq Hot-Start DNA Polymerase kit (Invitrogen™, Waltham, MA, USA). Then, 5 uL of cDNA was mixed with 4 uL 10x buffer, 0.4 uL of dNTP as well as each primer, 0.16 uL Taq, and 9.64 molecular grade water. Thermal cycling parameters were as follows: initial denaturation at 94 °C for 2 min; 35 cycles of denaturation at 94 °C for 15 s, annealing at 50 °C for 15 s, and elongation at 68 °C for 15 s. The product (2 uL) was used as template for a second-round of amplification with 10 uM of each primer: SARS2_S_F2 (CCCCTGCATACACTAATTCTT) and SARS2_S_R2 (AAACTTCACCAAAAGGGCACAAG). The reaction volumes were the same as in the first round with a total reaction volume of 20 uL. Conditions were also the same except annealing at 55 °C and resulted in a 900 kb product. PCR products were purified and sequenced at Inqaba Biotec, South Africa.

### 2.6. Phylogenetic Analyses

The ‘Phylogenetic Assignment of Named Global Outbreak Lineages’ (PANGOLIN) software suite (https://github.com/hCoV-2019/pangolin (accessed on 21 July 2021)) was used for SARS-CoV-2 lineage classification [31]. Outbreak strains were compared to global Delta sequences collected between May and June 2021. The 43,322 sequences were subsampled to 1545 in Nextstrain based on genetic proximity to the study sequences [32]. Sequences were aligned in ViralMSA and a maximum likelihood tree was inferred with IQ-TREE and the GTR + G4 model [33,34]. To determine the time to the most recent common ancestors of the study strains, a maximum likelihood (IQ-TREE) tree was inferred from 115 South African Delta sequences. TempEst v1.5.3 was used to plot the root-to-tip genetic distance and sampling dates and sequences that did not conform to a linear evolutionary pattern were removed from the dataset [35]. Bayesian phylogenetic inference was done in BEAST v1.10.5 using the GTR + G4 substitution model under a relaxed clock and coalescent Gaussian Markov random field (GMRF) [36]. A 100 million steps for a Markov chain Monte Carlo chain was run and every 10,000th generation was sampled. This was repeated twice. A maximum clade credibility (MCC) tree was summarized using TreeAnnotator v1.10.5. Trees were visualized and annotated using the FigTree (v1.4) program and Microreact [37].

## 3. Results

### 3.1. Clinical Features of SARS-CoV-2 Infection in Pumas and Lions

On 19 July 2020 one 12-year-old female puma (LPZ0018) in a private zoo in Johannesburg, Gauteng province of South Africa, showed signs of anorexia, followed 24 h later by a second puma (LPZ0017) with similar signs (Figure 1A). Nearly a year later, on the 12 June 2021, two lions (ZRU127/21 and ZRU128/21) became sick with nasal and ocular discharge and coughing in the same private zoo. Four days later, a 14-year-old female lion (ZRU125/21) housed in a separate enclosure also became sick (Figure 1A). This lioness developed lower respiratory tract infection with signs of bronchial pneumonia (Figure 1B). These animals were unresponsive to antibiotic therapy. NP samples from one puma (LPZ0018) and all three lions tested positive for SARS-CoV-2 by real time reverse transcriptase polymerase chain reaction (RT-PCR). Upon diagnosis, LPZ0018 was treated with doxycycline intramuscular (Bayer Animal Health, South Africa), a non-steroidal anti-inflammatory drug (meloxicam, 0.05 mg/km subcutaneously, Boehringer Ingelheim, South Africa) and a vitamin supplement (0.1 mL/kg, Kyroligo, Kyron Laboratories, South Africa). This resulted in an improvement in condition despite NP samples remaining positive for 4 weeks on faecal swabs and 6 weeks on nasal swabs. The two lions, ZRU127/21 and ZRU128/21, were initially also placed on oral antibiotics and non-steroidal anti-inflammatory drugs (Meloxicam) (Figure 1A). ZRU125/21 was treated with a single dose of dexamethasone (Kortico, Bayer Ltd., Johannesburg, South Africa) and antibiotics (Draxxin, Tulrathromycin, 200 mg SC, Zoetis, South Africa) for secondary bacterial infection. All three lions fully recovered approximately 3 weeks post disease onset. The lions were placed under quarantine from the time they tested positive until they were cleared from infection (7 weeks post onset of signs).

### 3.2. Kinetics of SARS-CoV-2 Infection

**Figure 2:**
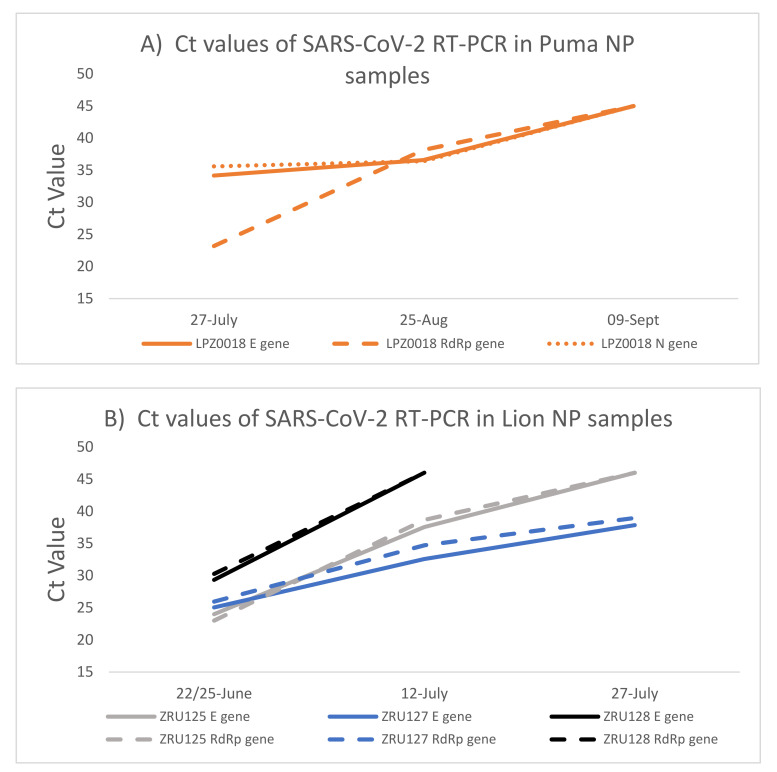
SARS-CoV-2 RNA load in puma and lions measured by RT-PCR over time. (**A**): Ct values detected in NP samples of the puma (LPZ0018). (**B**): Ct values detected in NP samples of the three lions. NP—Nasopharyngeal swab; E—SARS-CoV-2 envelope gene (solid lines); RdRp -SARS-CoV-2 RNA dependent RNA polymerase gene (dashed lines); N—SARS-CoV-2 nucleocapsid gene (dotted lines).

The puma and the lions were monitored for 10 weeks (puma) and 5 weeks (lions) after initial identification of SARS-CoV-2 infection. Both NP (Figure 2) and faecal samples (Supplementary Table S1) were used for RT-PCR testing. Except for the RdRp target (Ct: 23.2), the Ct values for the puma (LPZ0018) infection were already high (E gene Ct: 34 and N gene Ct: 36) when the first sample was taken, suggesting that the animal was towards the end of acute infection. Ct values in the mid-30s across all targets were still present after 1 month (6 weeks after initial signs) (Figure 2A). The virus was undetectable by 9 September 2020. Due to the low level of viral RNA, we were unable to use this sample for whole genome sequencing. The second puma (LPZ0017) only had mild clinical signs and no cough and thus it was decided not to anaesthetise the animal at that time for testing.

Two of the lions, ZRU125/21 and ZRU127/21, exhibited similar infection kinetics with a peak of viral RNA load detected (E gene Ct: 24 and 25) (Figure 2B) at the first collection point. ZRU128/21 showed lower levels of viral RNA at this same time point (Ct: 29.3). Less RNA was detected as the lions recovered from disease; however, viral RNA could still be detected in the nasopharynx of ZRU127/21 5 weeks (27 July 2021) after the first sample was taken (Ct: 37.84) i.e., 7 weeks after onset of clinical signs.

Lion faecal samples tested positive for 3 weeks after clinical signs appeared. No faecal samples tested PCR positive after 7 July 2021. This suggested that the lions were clear of viral RNA in their faeces approximately 1 month after first signs (Supplementary Table S2). No viral RNA was detected in the blood. In combination, these data show that SARS-CoV-2 infected and replicated in the respiratory and gastrointestinal tract in at least one puma and all three lions, concomitant with symptomology that is similar to COVID-19 in humans.

### 3.3. Investigation of Human–Lion Transmission Route

A One Health investigation into the source of infection to the lions was conducted on twelve members of staff who had either been in direct or indirect contact with the lions through structured interviews and collection of respiratory samples following informed consent. Both RT-PCR (NP/OP swabs) and ELISA (serum) testing were carried out (Supplementary Table S1). One staff member with direct contact (ZRUCWL005) and one with indirect contact (ZRUCWL012) tested PCR positive for SARS-CoV-2 on 25–26 June 2021 (approximately 2 weeks after the start of the lion disease course and while all three lions were PCR positive). These two individuals and three more staff members (a total of five staff members) also tested positive for anti-Spike IgG antibodies. None of the staff interviewed reported any recent symptoms of COVID-19. Follow-up samples were collected from the two PCR positive members of staff 17 days after the first tests. Both follow-up samples were still positive by PCR with Ct values of 33.30 (ZRUCWL005) and 35.95 (ZRUCWL012). These data suggest that SARS-CoV-2 was circulating amongst the staff during the time at which the lions got sick and suggests that staff members with direct contact with the lions were likely responsible for transmission.

### 3.4. Genomics of SARS-CoV-2 in the Lion Outbreak

**Figure 3:**
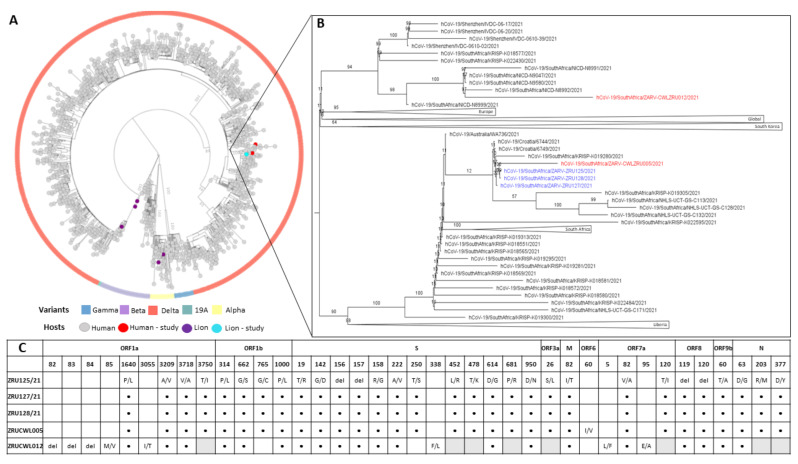
(**A**): Maximum likelihood tree of global SARS-CoV-2 genomes. Variants of concern are indicated in the outer circle and host species are indicated by coloured tips. (**B**): View of branch where SARS-CoV-2 sequences determined in this study clustered. Sequences from lions are in blue and sequences from humans are in red: ZRU125/21 (EPI-ISL-6261983), ZRU127/21 (EPI-ISL-6261987), ZRU128/21 (EPI-ISL-6261989), ZRUCWL005 (EPI-ISL-6261993) and ZRUCWL012 (EPI-ISL-6261996). (**C**): Amino acid changes in study strains when compared to the Wuhan-Hu-1 reference genome (NC_045512.2). Black dots represent identical changes and grey boxes represent missing data. Sequencing was done from NP swabs.

In order to determine if the staff and lions were infected with the same strain and to shed light on the route of transmission, genome sequencing was conducted on both humans and the three lions. We obtained near full-length sequences (92.3–98.4%) for all five samples with gaps in the Spike gene filled in by sanger sequencing. All five sequences had between 99.93 and 100% nucleotide identity. NextClade analysis as well as multiple sequence alignment (MSA) of the Spike glycoproteins revealed that each of the infections was a Delta variant (B.1.617.1) of SARS-CoV-2. More specifically, the three lions and ZRUCWL005 were classified as the AY.38 lineage of Delta (Figure 3). Phylogenetic analysis comparing the study sequences with local and global strains confirmed that all three lions and the keeper, ZRUCWL005, clustered together with South African sequences while ZRUCWL012 clustered in a separate South African clade (Figure 3). Additionally, the SARS-CoV-2 sequences detected in the South African lions were divergent from the Delta sequences detected in India from an outbreak in April/May 2021 [25]. Bayesian analysis indicated that the time of the Most Recent Common Ancestor (tMRCA) between the lions and ZRUCWL005 was around the end of May (95% HPD 2021.35-2021.45) and clustered with sequences detected in Gauteng, KwaZulu Natal, and Limpopo provinces of South Africa (Supplementary Figure S1). ZRUCWL012 shared a MRCA with the rest of the study strains around the middle of April (95% HPD 2021.15-2021.32).

## 4. Discussion

The results presented in this study document outbreaks of SARS-CoV-2 in pumas and lions kept in a South African private zoo. The two pumas and three lions presented with respiratory illness which was similar to COVID-19 in humans. Clinical signs in the animals in this report ranged from mild influenza-like illness including cough, to difficulty breathing and pneumonia. Additionally, both pumas and the three lions presented early on with ocular and/or nasal discharge, a sign that may be distinctive from human infection. The animals did not respond to antibiotic treatment but made uneventful recoveries following treatment with anti-inflammatory drugs and supportive care. Cases reported in large felids in this study are considered mild and are similar to mild infections in 32 confirmed positive large felids housed in zoological collections from April 2020 to August 2021 [38].

Detection of viral RNA in both the upper respiratory tract and the faeces as well as the fact that the pumas and the lions presented with the concomitant symptoms illustrates that this virus is able to generate a bona fide infection within these animals via a natural infection route. Despite extended viral shedding, all of the infected cats recovered fully. These outbreaks are at least the third and fourth of its kind in which SARS-CoV-2 has been shown to transmit between humans and captive large felines, although the current study is the only to report on genomic One Health investigations of Delta variants transmitted from humans to animals. These reports, as well as the evidence of experimental infection, make it clear that large felids are particularly susceptible to this virus.

Unfortunately, we were unable to carry out an investigation into the source or the specific variant involved in the puma outbreak. The samples were diagnosed by real-time PCR at the time of the outbreak, but when we attempted to sequence the sample a year later there was insufficient RNA left for genome sequencing. A One Health epidemiological investigation on the lion outbreak indicated that two staff members of the zoo also had SARS-CoV-2 in 2021. Whole genome sequencing and phylogenetic analysis indicated that ZRUCWL005, detected in a staff member who had direct contact with the lions, was closely related to the lion sequences. The genome sequence of the second staff member (ZRUCWL012) who did not have direct contact with the lions was slightly divergent and seems not to be part of the reverse zoonotic outbreak. There were little to no differences in nucleotide identity (99.93–100%) between the lion sequences and ZRUCWL005. This indicates that unlike in mink and in this case, a host switch did not result in evolutionary pressure to change the sequence of the SARS-CoV-2 variant [21] although only early sequences were obtained from the lions.

**Figure 4:**
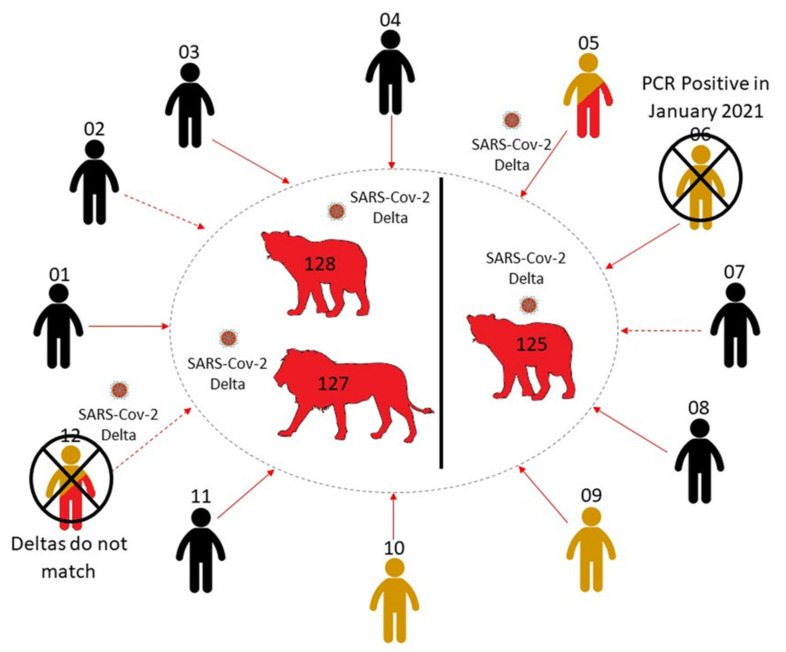
A summary of the potential infection route from animal handlers to the three lions. Direct (solid line) and indirect (dashed line) human contacts were traced and tested for SARS-CoV-2 RNA and IgG antibodies. Negative cases are coloured in black. PCR positive cases are coloured in red, while serologically positive cases are coloured in yellow. The two contact cases which were unlikely to be responsible for infecting the lions, owing to differing Delta sequences (ZRUCWL012) and previous positive tests (ZRUCWL006) are marked with a cross.

The timeline of infections for lions and ZRUCWL005 is difficult to estimate since all staff members were asymptomatic during the outbreak. RT-PCR results from the study might indicate that the lion and human infections occurred more or less in parallel. This leads us to believe that the transmission route for this outbreak is either ZRUCWL005 (the head big cat keeper) or another human contact, although the true direction of transmission is difficult to estimate. Three other staff members tested positive for anti-Spike IgG antibodies and only one reported a previous known positive COVID-19 test. This result was in January 2021, which was too long before the outbreak to be related. It is therefore also possible that one of these staff members infected ZRUCWL005 and the lions concomitantly (Figure 4). The index infection was likely in May when these sequences shared a MRCA. Since ZRU127/21 and ZRU128/21 presented with clinical signs on the same day, it is likely that each was infected by the original source. It is also possible that the original index case, whether identified or not in this study, transmitted the virus to the lions which subsequently passed it on to ZRUCWL005. Isolation of infectious virus from lion swabs was inconclusive and it was not possible to determine whether the lions were shedding infectious virus at the time of sampling. It is, however, clear that at least two reverse zoonotic events occurred in June 2021 in this zoo since ZRU125/21 was kept in a separate cage with no contact to the other two lions.

Reverse zoonotic transmission of SARS-CoV-2 from asymptomatic animal handlers pose a risk to large felines kept in captivity. Transmission of the Delta variant to these animals may potentially result in more severe disease. Prolonged shedding may spread the virus to animals in close proximity. Precautions should be implemented in zoos and other settings where these animals may have frequent exposures to humans to prevent such events and in particular to avoid introduction of SARS-CoV-2 to the wider population of animals in the wild where control measures are difficult to implement sufficiently early.

## Data Availability

Whole genome sequences were uploaded to GISAID with the following accession numbers: ZRU125/21 (EPI-ISL-6261983), ZRU127/21 (EPI-ISL-6261987), ZRU128/21 (EPI-ISL-6261989), ZRUCWL005 (EPI-ISL-6261993) and ZRUCWL012 (EPI-ISL-6261996).

## Supplementary Materials

The following are available online at https://www.mdpi.com/article/10.3390/v14010120/s1, Table S1: PCR and serological results from direct and indirect human contacts with infected lions, Table S2: Detection of SARS-CoV-2 E and RdRp genes from lion faecal samples in Ct values, Figure S1: Bayesian phylogenetic inference of whole genome sequences detected in lions and humans.
