# Supplementary material for: SARS-CoV-2 Reverse Zoonoses to Pumas and Lions, South Africa
Source: Viruses. 2022 Jan 11;14(1):120. doi: 10.3390/v14010120 (PMC8778549; doi:10.3390/v14010120)
Supplement: Supplementary file 1 [file viruses-14-00120-s001.zip › viruses-1529694-supplementary.pdf]

Suppl Table S1: PCR and serological results from direct and indirect human contacts with infected lions.

	**PCR**		**Serology**				
**ID**	**Result (Ct value)**	**Date of result**	**Result**	**Date of Result**	**Job at Zoo**	**Nature of Contact with Animals**	**Previous COVID-19 test**
ZRUCWL001	Negative	25/06/2021	Negative	25/06/2021	Keeper	**Direct:** Enter enclosure, contact with food and waste	None
ZRUCWL002	Negative	25/06/2021	Negative	25/06/2021	Kitchen staff	**Indirect:** Handle food/utensils	None
ZRUCWL003	Negative	25/06/2021	Negative	25/06/2021	Cats Keeper	**Direct**: Enter enclosure, contact with food and waste	None
ZRUCWL004	Negative	25/06/2021	Negative	25/06/2021	Owner	**Direct:** Touching	None
**ZRUCWL005**	**Positive (27,26; 33,3)**	25/06/2021 12/07/2021	**Positive**	25/06/2021 12/07/2021	Head big cat keeper	**Direct:** Enter enclosure, contact with food and waste	None
**ZRUCWL006**	Negative	25/06/2021	**Positive**	25/06/2021	Manager	**Direct:** Enter enclosure, contact with food and waste/Darting	Yes (positive) 04/01/2021
ZRUCWL007	Negative	25/06/2021	Negative	25/06/2021	Kitchen staff	**Indirect:** Handle food/utensils	None
ZRUCWL008	Negative	25/06/2021	Negative	25/06/2021	Assistant manager	**Direct:** Enter enclosure, contact with food and waste/Darting	None
**ZRUCWL009**	Negative	25/06/2021	**Positive**	25/06/2021	Big cat keeper	**Direct:** Enter enclosure, contact with food and waste	None
**ZRUCWL010**	Negative	25/06/2021	**Positive**	25/06/2021	Cubs keeper	**Direct:** Enter enclosure, contact with food and waste/Darting	None
ZRUCWL011	Negative	25/06/2021	Positive (Vaccinated)	25/06/2021	Veterinarian	**Direct:** Direct contact during medicals/darting	None
**ZRUCWL012**	**Positive (ND; 23,28)**	26/06/2021 12/07/2021	**Positive**	25/06/2021	Maintenance	**Indirect**: Maintenance	None

Suppl Table S2. Detection of SARS-CoV-2 E and RdRp genes from lion faecal samples in Ct values.

	Target	25-Jun-21	07-Jul-21	10-Jul-21	12-Jul-21
**ZRU125/21**	E	No sample	33,5	Neg	Neg
	RdRp	No sample	Neg	Neg	Neg
**ZRU127/21**	E	37,53	Neg	Neg	Neg
	RdRp	Neg	Neg	Neg	Neg
**ZRU128/21**	E	32,73	Neg	Neg	Neg
	RdRp	34,7	Neg	Neg	Neg

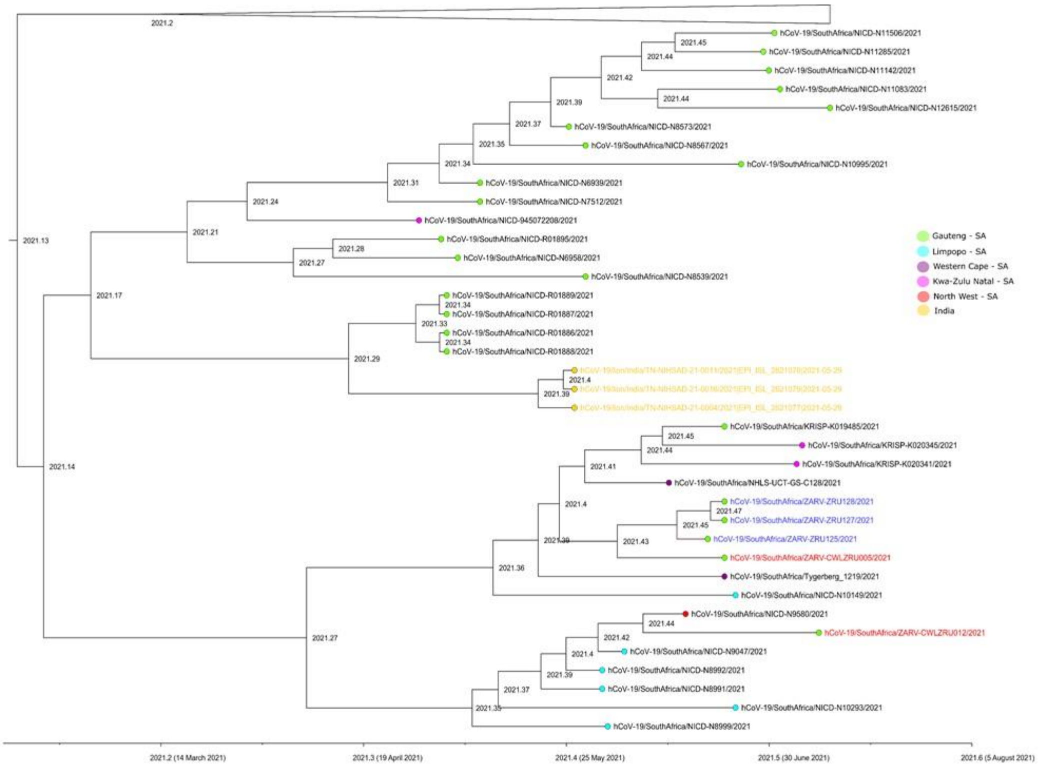

Suppl Figure S1. Bayesian phylogenetic inference of whole genome sequences detected in lions and humans. Node tips are coloured by area where samples were taken. Lion samples detected in India is coloured in yellow. Study samples are coloured in blue (lions) and red (humans); ZRU125/21 (EPI-ISL-6261983), ZRU127/21 (EPI-ISL-6261987), ZRU128/21 (EPI-ISL-6261989), ZRUCWL005 (EPI-ISL-6261993) and ZRUCWL012 (EPI-ISL-6261996).
